# A Comprehensive Study on Measurement Accuracy of Distributed Fiber Optic Sensors Embedded within Capillaries of Solid Structures

**DOI:** 10.3390/s23198083

**Published:** 2023-09-26

**Authors:** Yuzhe Xiao, Calvin Rans, Dimitrios Zarouchas, Rinze Benedictus

**Affiliations:** Faculty of Aerospace Engineering, Delft University of Technology, Mekelweg 5, 2628 CD Delft, The Netherlands

**Keywords:** fiber optic sensor embedding, strain measurement, structural health monitoring, measurement accuracy

## Abstract

Embedding fiber optic sensors (FOSs) within parts for strain measurement is attracting widespread interest due to its great potential in the field of structural health monitoring (SHM). This work proposes a novel method of embedding FOSs using capillaries within solid structures and investigates fiber positions and orientation uncertainties within capillaries of different sizes and their influences on strain measurement accuracies. To investigate how the fiber positions and orientation variations influence strain measurement accuracy, both analytical and numerical models are utilized to predict strain distributions along embedded fibers at different positions and with different orientations within the specimen. To verify the predictions, a group of specimens made of Aluminum 6082 was prepared, and the specimens in each group had capillaries of 2 mm, 4 mm, and 6 mm diameters, respectively. Fibers were embedded within each specimen using the capillaries. Four-point bending static tests were conducted for each specimen with embedded FOSs, performing in situ strain measurement. Subsequently, the specimens were partitioned into several pieces, and the cross sections were observed to know the real positions of the embedded fiber. Finally, the strain predictions at the real locations of the fiber were compared with the measured strain from the embedded FOSs. The predicted strain distributions as a function of the fiber positions alone and as a function of both the fiber positions and orientations were compared to assess the influence of fiber orientation change. The results from a combination of analytical, numerical, and experimental techniques suggest that the fiber position from the capillary center is the main factor that can influence strain measurement accuracies of embedded FOSs, and potential fiber misalignments within the capillary had a negligible influence. The fiber position-induced measured error increases from 10.5% to 18.5% as the capillary diameter increases from 2 mm to 6 mm. A 2 mm capillary diameter is able to lead to the lowest measurement error in this study and maintains ease of embedding. In addition, it is found that the measured strain always lies within a strain window defined by the strain distribution along capillary boundaries when there are no cracks. This can be further studied for crack detection.

## 1. Introduction

Structural health monitoring (SHM) is a process aimed at providing accurate and timely information concerning structural health condition and performance. An SHM system can perform real-time monitoring and makes non-destructive testing an integral part of structures, which can reduce maintenance costs and increase structural life safety in aerospace and civil engineering [1]. Sensors can be mounted on structures externally or embedded within structures. Both internal and external sensors can be utilized for strain measurements, but there are growing cases where only internal sensors are applicable. For example, when knowledge of mechanical signals inside the structure is needed, sensors bonded on the outside can no longer be used. In addition, the embedded sensor can demonstrate stronger applicability for structures functioning in harsh conditions, such as corrosive environments, which may disable external sensors [2]. The need for information inside the structure in sectors including aerospace [2] demands reliable technology for embedding sensors within structures. There are two main approaches to embedding a sensor within a structure. It can be embedded concurrently with the manufacturing process, or the sensor can be embedded post-manufacturing. There is a large base of experience documented in the literature on embedding optical fibers within composite structures. The general approach is to integrate the fiber within a composite layup concurrently with the manufacturing process. Although there are a number of variations of this, including placing optic fibers between individual pre-preg plies [3], co-braiding fibers into braided composites [4] and weaving optical fibers within the composite fabric [5], these processes are only applicable to composites. There are fewer examples where fibers are embedded concurrently with the additive manufacturing (AM) process [6,7,8,9,10]. In these cases, the AM process is typically interrupted, and fibers are placed within the build plane, and subsequent continuation of the layer-by-layer manufacturing is resumed over the placed sensor. Hyer et al. used the ultrasonic to embed fiber optics in soft materials, such as Al and Cu, by placing the sensors in machined cavities and ultrasonically welding over the top with thin foils [11]. However, this introduces an issue, where the high temperature during the embedding process can induce coating degradation or even thermal damage of the fiber. The annealing point of fused silica (around 1100 °C) is low when compared to the melting point of Ti6Al4V (around 1604 °C) [6]. A common approach to tackling the problem is to protect the fiber with metal jackets before fiber embedding; however, this makes the embedding process complex and carries the risk of introducing bonding defects between the fiber and host material. An example of the difficulty is highlighted by the work of Havermann [6], where the building process of selective laser melting (SLM) is interrupted, a fiber is inserted into a groove, and encapsulation of the fiber is performed by continuing the SLM process. Havermann found that bonding imperfections remain between the fibers and host material due to lack of fusion. Specifically looking at the fiber coating process, Li investigated the application of the metal coating through electro-plating and magnetron sputtering, both adding more complexity and cost to the fabrication process [7]. A number of other coating and embedding methodologies have been studied, including an electrodeless- electro nickel plating of an optical fiber [8], vacuum brazing of nickel coated fiber by Sandlin [9], and ultrasonically welding a metal-coated FBG sensor into an Aluminum 6061 part by Schomer [10]. In the end, the cost and complexity, combined with the limited placement freedom to a single build plane make the fiber-embedding approach quite limiting, and motivate the investigation of post-manufacturing embedding strategies within this paper.

With the development of novel manufacturing technologies, new fiber-embedding techniques are emerging. Additive manufacturing (AM) featuring layer-wise deposition of layers can be used in fiber embedding. Strantza [12] fabricated a vacuum sensor by integrating a capillary within a part using SLM, and demonstrated it to be effective in detecting edge cracks. As this sensor type relies on cracks growing up to the embedded capillary in order to disrupt its vacuum condition, the capillary needs to be placed in close proximity to fatigue hot spots in order to be effective. In such a location, the capillary itself can pose a risk of acting as a stress raiser for initiating fatigue damages. Despite this limitation, the concept of capillaries within a structure can be explored for other sensing concepts. One possibility is to combine the capillary concept proposed by Strantza [12] with FOSs for SHM. Specifically, functional parts can be manufactured with pre-defined capillaries into which sensing fibers are inserted and bonded afterward. The embedding process is potentially easier compared to the process where the manufacturing process is interrupted, and provides the most freedom with respect to the path that a fiber can take (it would not be limited to a single build plane).

**Figure 1:**
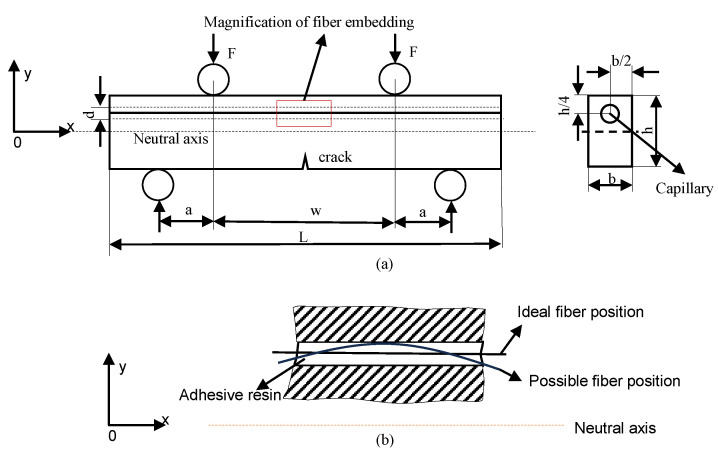
(**a**) The schematic representation of the specimen under four-point bending; (**b**) magnification of the embedded fiber at the ideal position and other possible position.

However, some challenges can still be expected from this fiber-embedding technique. When a fiber is embedded into a specimen using capillaries, because the diameter of a capillary is normally larger than that of a fiber for ease of embedding, the fiber may not be placed at an exact position. Ideally, the fiber is placed along the capillary center; however, it could be anywhere within the capillary in reality as shown in Figure 1. The fiber positions and orientations uncertainty could also lead to strain measurement uncertainty. The measured strain distributions will be different from the strain distributions along the capillary center in an ideal situation. The differences bring up an issue of strain measurement accuracy. This paper aims to study the position and orientation variations of the fiber embedded into specimens using capillaries, how fiber positions and orientations variations influence strain measurement accuracy for specimens, and the range of the measured strain distribution. A previous study [13] by the authors performed preliminary research on the measurement accuracies by comparing the analytical results and test results, and this study improves it by refining the analytical modeling process and using a combination of analytical, numerical, and experimental techniques.

## 2. Methodology

The main objective of this research paper is to investigate the influence of possible variations in the position and orientation of a distributed sensing fiber placed within a predefined capillary on strain sensing accuracy. Geometries of used specimens are simplified to a beam structure, and four-point bending tests are performed, as shown in the Figure 1. There are two reasons for adopting the specimen geometry and four-point bending tests. The first one is that the specimen geometry is the same as that in a previous study [11] focusing on crack detection with vacuum sensors in SLM specimens, so adopting the same specimen geometry facilitates a comparison of the performance of the proposed crack detection approach with the previous study. Secondly, the four-point bending can create a region with a constant bending moment between the two loading pins. In the region, the influence of the fiber locations and orientations can be highlighted, and the influence of bending moment does not need to be considered. Traditionally machined specimens will be used for testing. Existing defects in additively manufactured specimens could potentially affect the strain measurement accuracy. Using the traditionally machined specimens can avoid the possible influence of defects in the additively manufactured parts, and emphasize the influence of the pre-manufactured capillary on the measurement accuracy. The latter is the focus of this study.

**Figure 2:**
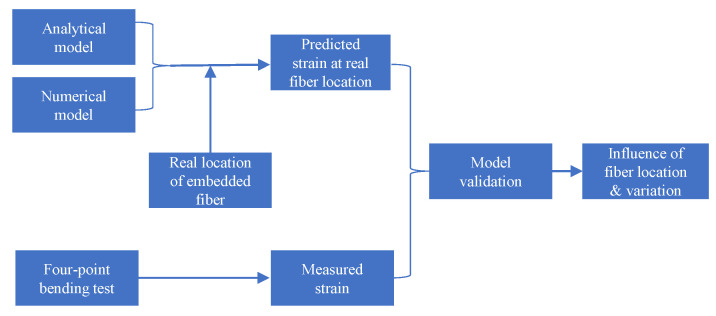
Overview of the methodology.

An analytical model based on the Euler–Bernoulli beam theory was developed to predict strain distribution along the embedded fiber, which is assumed to be along the capillary center shown as the ideal position of fiber in Figure 1. The Euler–Bernoulli beam theory assumes that the beam under bending is sufficiently slender (i.e., has a slenderness ratio (length to thickness) greater than 10); however, the specimen in this study has a slenderness ratio slightly less than 10. The reason for adopting the specimen geometry is that the specimen geometry in this study is the same as that in a previous study [11], focusing on crack detection with vacuum sensors in SLM specimens. Using the same specimen geometry facilitates a comparison of the performance of the proposed crack detection approach with the previous study. Therefore, a finite element model was created to predict the strain distribution at the same location as in the analytical model. In order to verify the predictive models, static four-point bending tests were performed on the Aluminum 6082 parts with embedded FOSs under 8 KN. After the four-point bending test, the specimen was cut to observe the real location of an embedded fiber in a capillary, and the predicted strain distribution along the embedded fiber was updated with the real location of the embedded fiber. For verifying the predictive model, the measured strain distribution from the embedded FOSs was compared with the predicted strain distribution at the real fiber location. An overview of the process can be seen in Figure 2.

### 2.1. Reference Specimen Geometry

In order to study the potential influence of sensor position variations on measurement accuracy, a reference structural geometry was needed. As the distributed sensing fibers only measure normal strains, a simple beam under four-point bending loading is a logical choice, as it creates a region along the beam where normal strains should not vary in the length direction. This would permit the observed scatter in strain measurements along the length of a FOS from later experiments to be more easily correlated to variation in its position and orientation. The reference geometry used in this study is illustrated in Figure 1, where *b*, *h*, *L*, and *d* denote the specimen width, height, length, and diameter of the capillary, respectively. The spacing *a* of the load introduction points results in a constant internal bending moment, along the central part of the beam denoted by *w*. The dimensions of the specimen are given parametrically, as the analytical model for this study was set up in parametric form.

### 2.2. Strain Distribution Prediction with a Simplified Analytical Model

In order to investigate the influence of the fiber position and orientation on the expected strain, a simple analytical model was made. The model focused on the central portion of the specimen (denoted by width *w* in Figure 1) where the internal moment was constant. Along this section, the normal stress due to bending, $\sigma_{x}$, can be described using Euler–Bernoulli beam theory [14]:

(1) $$\sigma_{x}=\frac{My}{I},$$

where *M* is the bending moment and is given by M=Fa, *F* is the force exercised on each of the loading, *a* is the distance between the upper and lower loading pins as shown in Figure 1, *y* is the distance from the neutral axis, and *I* is the moment of inertia around the neutral axis. As this section of the specimen was under pure bending loading, there are no other stress components, allowing the normal strain $\varepsilon_{z}$ to be calculated simply by dividing the stress by Young’s modulus *E*:

(2) $$\varepsilon_{x}=\frac{\sigma_{x}}{E}=\frac{My}{EI},$$

The Euler–Bernoulli bending theory in the form given in Equation (1) is only applicable for beams that are sufficiently slender (i.e., have a slenderness ratio greater than 10). For the reference specimen geometry, the symmetric section requirement is met; however, depending on the precise dimensions of the beam, the slenderness ratio is not met. This will be revisited later when discussing the experimental portion of the study.

**Figure 3:**
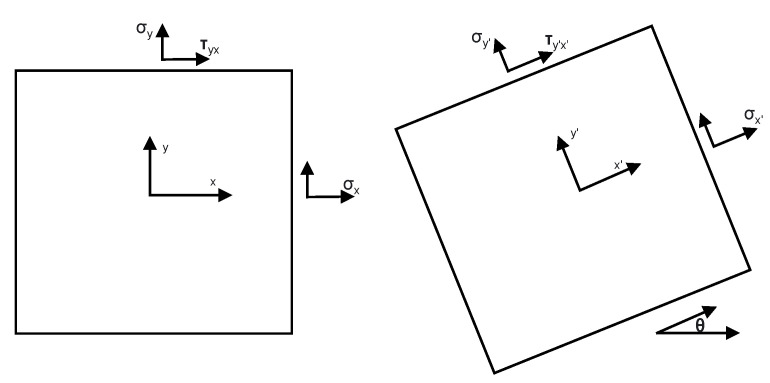
Stress transformation for a plane inclined at an angle.

In order to apply Equation (2) to a fiber of arbitrary position and orientation within a capillary, a correction for orientation must be applied. Considering a rotated coordinate frame $y^{\prime }-x^{\prime }$ (as illustrated in Figure 3), the normal stresses along the $y^{\prime }$ and $x^{\prime }$ directions can be given by the plane–stress transformation equation:

(3) $$\sigma_{x^{\prime }}=\frac{\sigma_{x}+\sigma_{y}}{2}+\frac{\sigma_{x}-\sigma_{y}}{2}cos\left(2\theta \right)+\tau_{yx}sin\left(2\theta \right),$$

(4) $$\sigma_{y^{\prime }}=\frac{\sigma_{x}+\sigma_{y}}{2}-\frac{\sigma_{x}-\sigma_{y}}{2}cos\left(2\theta \right)+\tau_{yx}sin\left(2\theta \right),$$

For the four-point bending specimen, the normal stress in the *y* direction $\sigma_{y}$ was zero. The loading is directly applied on the substrate structure, which is the specimen. The substrate structure and adhesive resins have different stiffness. Therefore, the shear stress exists in the adhesive resins within the capillary. Thus, the stress transformation of Equations (3) and (4) can be simplified to the following equation:

(5) $$\sigma_{x^{\prime }}=\frac{\sigma_{x}}{2}\left(1+cos\left(2\theta \right)\right)=\frac{\sigma_{x}}{1+tan{\left(\theta \right)}^{2}}+\tau_{yx}sin\left(2\theta \right),$$

(6) $$\sigma_{y^{\prime }}=\frac{\sigma_{x}}{2}\left(1-cos\left(2\theta \right)\right)=\frac{\sigma_{x}tan{\left(\theta \right)}^{2}}{1+tan{\left(\theta \right)}^{2}}+\tau_{yx}sin\left(2\theta \right),$$

Using Hooke’s law and the equations above, the normal strain in the $x^{\prime }$ direction can be obtained by

(7) $$\epsilon_{x^{\prime }}=\frac{\sigma_{x^{\prime }}-\upsilon \sigma_{y^{\prime }}}{E},$$

(8) $$\frac{\sigma_{x^{\prime }}-\upsilon \sigma_{y^{\prime }}}{E}=\frac{\sigma_{x}\left(1-\upsilon tan{\left(\theta \right)}^{2}\right)}{E(1+tan{\left(\theta \right)}^{2})}+\frac{\tau_{yx}sin\left(2\theta \right)\left(1-\upsilon \right)}{E},$$

(9) $$\epsilon_{x^{\prime }}=\frac{\sigma_{x}\left(1-\upsilon tan{\left(\theta \right)}^{2}\right)}{E(1+tan{\left(\theta \right)}^{2})}=\frac{My(1-\upsilon tan{\left(\theta \right)}^{2})}{EI(1+tan{\left(\theta \right)}^{2})}+\frac{\tau_{yx}sin\left(2\theta \right)\left(1-\upsilon \right)}{E},$$

Using this Equation (9), the normal strain at an arbitrary position *y* and arbitrary orientation θ can be calculated for a symmetric beam subjected to pure bending.

**Figure 4:**
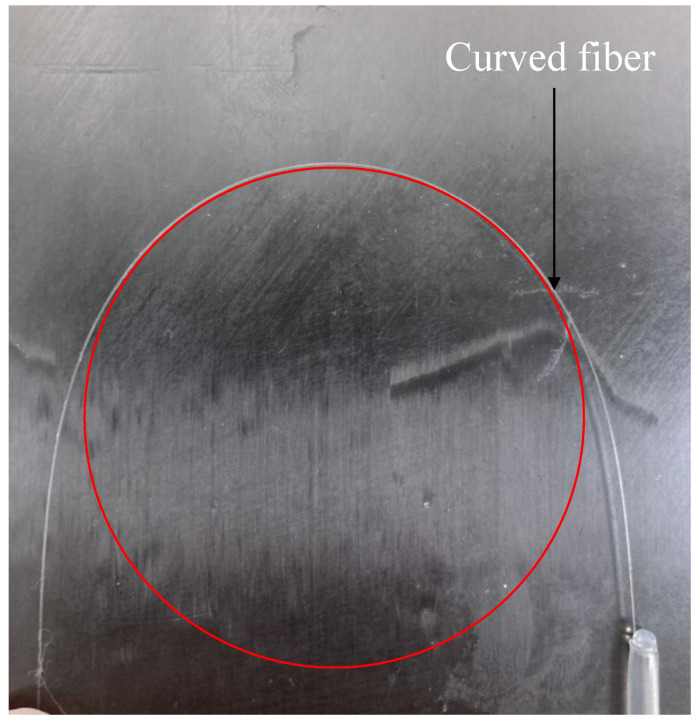
Arc-like shape of the curved fiber.

**Figure 5:**
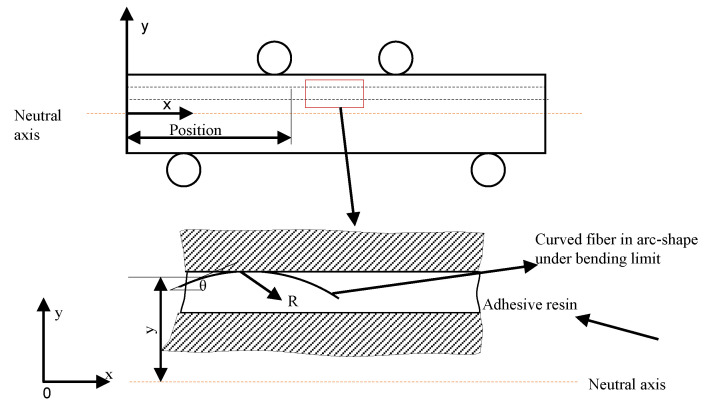
Arc-like shape of the curved fiber within the capillary.

Because the capillary size is much larger than that of the fiber, the embedded fiber can be at any location within the capillary, and any orientations can also be possible as long as it is within the fiber bending limit (bending limit of the fiber used in this study is 6 mm [15]). In addition, the curved fiber will take on an arc-like shape, depending on the degree of bending [16]. Figure 4 gives an example of the arc-like shape of a bending fiber used in this study. Therefore, the shape of the embedded fiber with curvature radius *R* within the capillary can be seen in Figure 5. To investigate the influence of the fiber position and orientation within the capillary, the fiber is modeled as an arc-like shape with different curvature radii. The variable *y* in this figure represents the distance from the neutral axis of the beam to the nominal position of the FOSs.

Using this model of the fiber location, the *y*-position ($y_{FOS}$) and tangent of fiber orientation ($\theta_{FOS}$) at any position *x* along the capillary can be calculated by

(10) $$y=\sqrt{R^{2}-{\left(x-\sqrt{2R-1}\right)}^{2}}-R+6,$$

(11) $$tan\theta \left(x\right)=\frac{\sqrt{2R-1}-x}{\sqrt{R^{2}-{\left(x-\sqrt{2R-1}\right)}^{2}}},$$

(12) $$sin\left(2\theta \left(x\right)\right)=\frac{2tan(\theta (x))}{1+tan^{2}\left(\theta \left(x\right)\right)},$$

Using Equations (9)–(12), the expected strain can be calculated along the axis of the embedded fiber with different shapes. When using Equation (9), one must be diligent in defining a suitable flexural rigidity term $EI_{xx}$. As the capillary will be filled with adhesive in practice, the cross section of the beam is a bi-material system, and the location of the neutral axis requires a stiffness-weighted centroidal position to be determined. However, within the current study, only two beam materials are considered: Aluminum 6082-T651 and a stereolithography resin. As the aluminum alloy is significantly stiffer than the adhesive in the capillary, the contribution of the adhesive to the flexural rigidity can be ignored, and it can be calculated by assuming the rectangular beam section with a hole in the location of the capillary.

### 2.3. Strain Distribution Prediction with a Finite Element Model (FEM)

The specimens tested in this study have a slenderness ratio which violates the assumption in the Euler–Bernoulli bending theory used in the analytical prediction model. Therefore, a numerical model was established using ABAQUS to investigate how much this violation may influence strain prediction accuracy.

#### 2.3.1. Material Model

The established model includes the specimens made of Aluminum 6082-T651 alloy and adhesive resins within its capillary. The material model is linearly elastic, and the material properties can be seen in Table 1. There are support pins defined with the discrete rigid type, which is assembled with the part to simulate four-point bending.

#### 2.3.2. Finite Element Model

**Figure 6:**
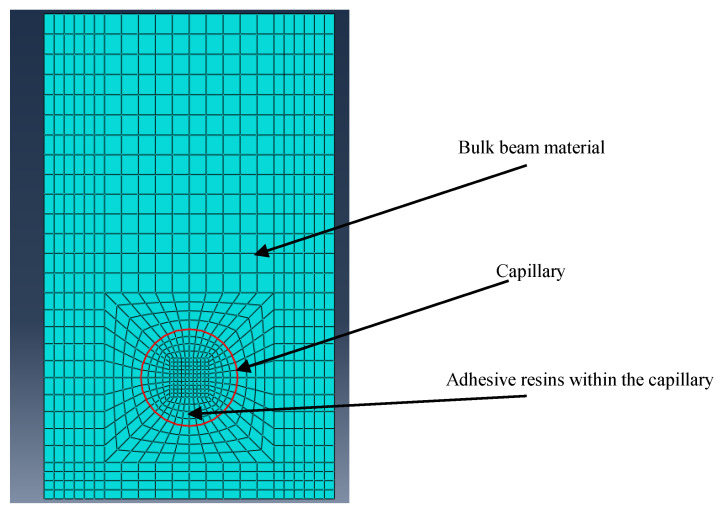
Illustration of mesh density around the interface between the adhesive resin-filled capillary and bulk beam material in the FEM model.

In order to model the bonding of the adhesive resin within the capillary of the specimen, a single part was divided into two pieces, each of which was assigned a separate section and material mentioned above. The two pieces represented the aluminum specimen with a capillary and the adhesive resin separately. Figure 6 presents the mesh both inside and outside a capillary. The mesh was designed with an increasing level of refinement from the center of the capillary to the outer aluminum alloy. It can be seen that there was a finer mesh around the adhesive resin and Aluminum 6082-T651 alloy interface, where a strain concentration may exist due to the material discontinuity. The element type is C3D8R. The smallest element is around the interface, which has a size of 0.15 mm. The biggest element size is at the region far from the interface, which has a size of 0.5 mm. The total number of elements in the model is 78,624. The element size near the interface was determined with a convergence study, and additional mesh refinement hardly affected the simulation results.

#### 2.3.3. Loading and Boundary Conditions

**Figure 7:**
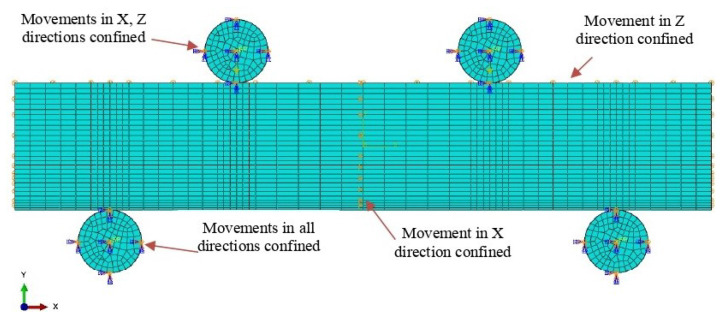
Boundary conditions for the four-point bending model.

The concentrated force was exercised on the upper pin supports. For boundary conditions, the lower two supports were constrained for all degrees of freedom. The upper pin supports were under a displacement/rotation constraint to limit their lateral movement along the X and Z directions. In addition, the movement of the central plan of the specimen along the X direction was confined. The boundary conditions can be seen in Figure 7. Surface-to-surface contact in the ABAQUS was exercised between the support pins and the model.

### 2.4. Verification of the Predictive Models

In order to investigate fiber position inaccuracy within the specimen capillaries, FOSs were embedded within specimens containing capillaries, and the specimens were subjected to four-point bending loading. Strain measurements from the fiber optic sensors were captured during loading, and post-mortem sectioning of the specimens was performed in order to accurately characterize the location of the FOSs within the capillary at various points along the beam length. Details of the manufacturing, testing, and post-mortem sectioning of the specimens are described in the remainder of this section.

#### 2.4.1. Specimen Fabrication

**Figure 8:**
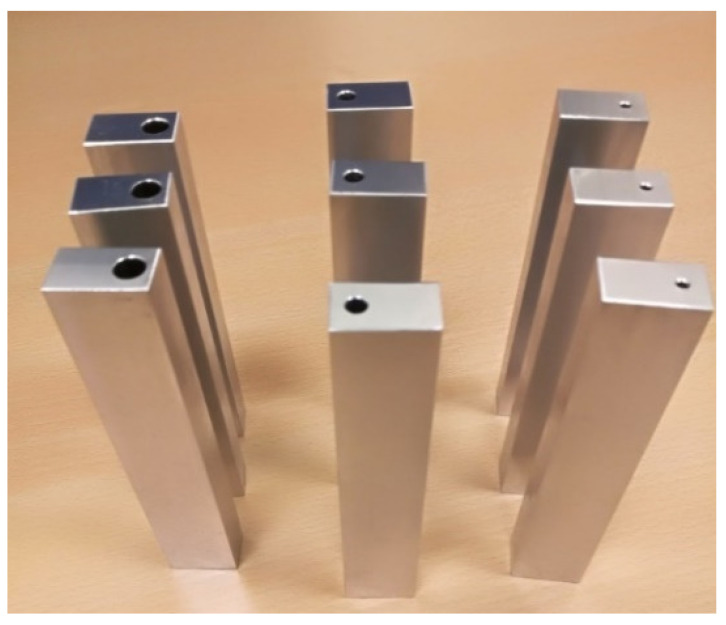
Aluminum 6082-T651 specimens with different sizes of capillaries.

Aluminum 6082-T651 specimens were fabricated using traditional subtractive machining processes. The use of subtractive manufacturing processes over additive manufacturing processes was chosen to avoid potential variations in the capillary and overall specimen geometry that are known to occur in metal additive manufacturing processes. Specimens with different sizes of capillary are provided in Figure 8, the details of all specimen configurations are provided in Table 2, and the meaning of different symbols can be seen in Figure 1.

After specimen fabrication, the following procedures were conducted to embed a fiber within the capillary of each specimen. First, a commercially available optical fiber (LBL-1550-125, FBGS, Geel, Belgium) was placed within the specimen capillary. The optical fiber has a cladding diameter of 125 με, and a coating diameter of 195 με. Second, the two-component epoxy adhesive (Araldite2011) was prepared by mixing resin of AW 106 and hardener of HV 953U by a weight ratio of 5:4. Third, the optical fiber was inserted into the capillary. This insertion process will be elaborated on later in this section. Finally, the prepared adhesive was injected into the capillaries slowly using a syringe. All the specimens with an embedded fiber were placed into an oven for resin curing; for Araldite 2011, this process normally takes 3 h under the temperature of 40 °C

#### 2.4.2. Four-Point Bending Test

**Figure 9:**
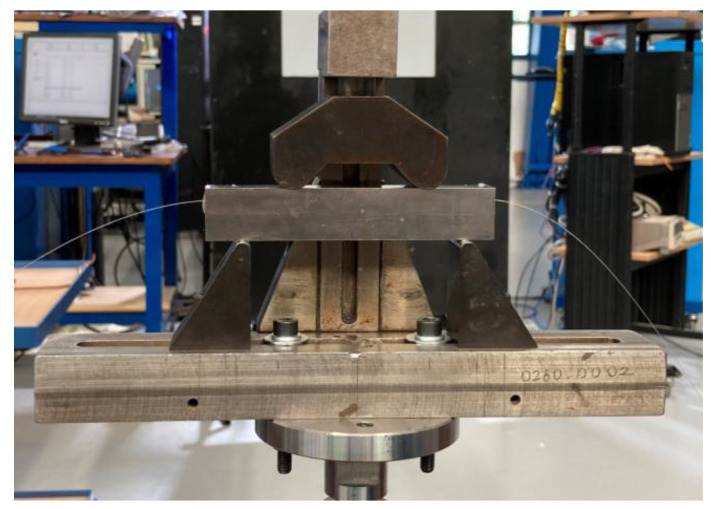
Experimental setup of a four-point bending test on Aluminum 6082-T651 specimens.

In order to evaluate the strain measurement accuracy of the embedded FOSs, all specimens were subjected to static loading in a four-point bending setup, which can be seen in Figure 9. The machine used is the Zwick-10kN tensile/compression machine. A static loading level of 8 KN was exercised on the Aluminum 6082 specimens quasi-statically. Different test cases can be seen in Table 3. During the four-point bending test, the FOSs were interrogated by the LUNA ODISI-B system to measure the strain distribution; these are compared with both the analytical and numerical results.

#### 2.4.3. Specimen Sectioning

The fiber-embedding process used in this study could not ensure an accurate fiber location within the capillary. As a result, after the testing of the specimens was completed, they were sectioned at various positions along the length of the specimen in order to assess the precise location of the optical fiber within the capillary. Sections of the specimens were observed with a laser microscope, which contained built-in software that could be used to precisely measure the location of the optical fiber within the capillary.

## 3. Results

### 3.1. Analytical Model Results

**Figure 10:**
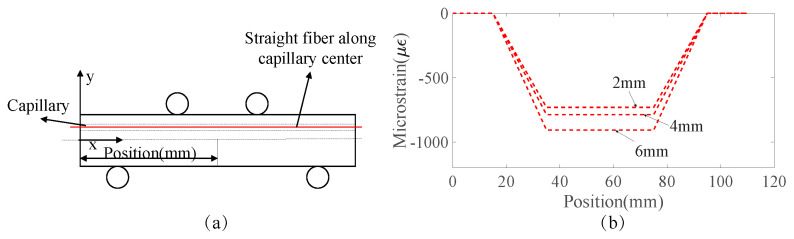
(**a**) Schematic representation of an embedded fiber which is straight and at the capillary center. (**b**) Analytically predicted strain distribution along the capillary center when the capillary diameters are 2 mm, 4 mm, and 6 mm, respectively, for Aluminum 6082 under the loading of 4 KN.

**Figure 11:**
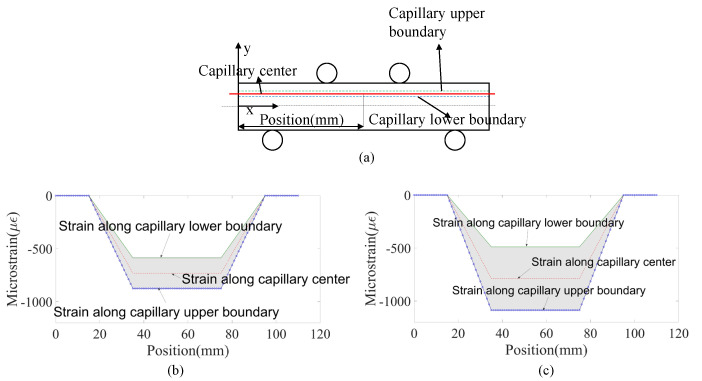
(**a**) Capillary upper boundary, capillary center, and capillary lower boundary in the specimen under four-point bending; analytically predicted strain distribution, which is along the straight fiber at the capillary center, upper boundary, and lower boundary when capillary diameters are (**b**) 2 mm and (**c**) 4 mm in Aluminum 6082 specimens under the loading of 8 KN.

Figure 10 and Figure 11 present analytically predicted strain distributions along a straight fiber within a capillary when the capillary diameters and fiber positions are different, which are predicted with the analytical model described in Section 2.2. The predicted strain distributions are negative due to the fact that the strain is the compressive strain. Figure 10 illustrates the analytically predicted strain distribution along a straight fiber at the capillary center when the capillary diameter varies from 2 mm to 6 mm. Figure 11 shows the analytically predicted strain along the capillary upper and lower boundary, which make up the strain window when the capillary diameters are 2 mm and 4 mm. The size of the strain window depends on the capillary diameters, and the strain window size increases as the capillary diameter increases from 2 mm to 4 mm.

### 3.2. Numerical Results

**Figure 12:**
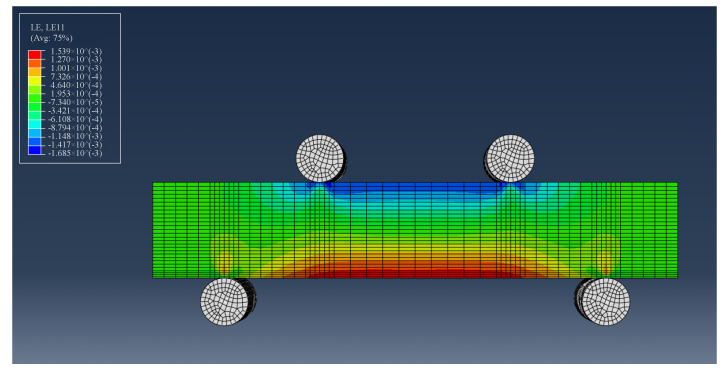
Numerically predicted strain distribution over the specimen under four-point bending.

**Figure 13:**
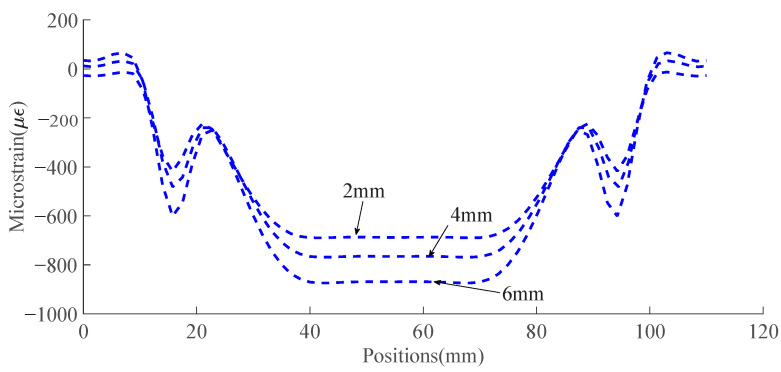
Simulated strain distribution along capillary centers when capillary diameters are 2 mm, 4 mm, and 6 mm, respectively, under the 8 KN loading.

Figure 12 presents the prediction of strain distribution over the specimen under four-point bending from the numerical model and Figure 13 represents strain distributions along the capillary centers when capillary diameters are 2 mm, 4 mm, and 6 mm, respectively.

### 3.3. Cross Sections of the Capillary with Embedded Fiber

**Figure 14:**
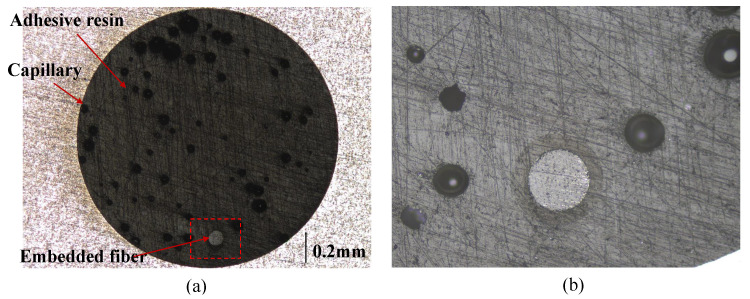
(**a**) Cross section of one specimen with the embedded fiber; (**b**) magnification of the region with embedded fiber.

A cross section of a 2 mm diameter capillary with straight fibers in the Aluminum 6082 specimen was observed using the Keyence Laser Scanning Confocal Microscope (DASML, Tudelft, The Netherlands), which is presented in Figure 14. The fiber was placed along the capillary center before injecting adhesives as described in Section 2.4.1. However, it can be seen that the embedded fiber deviates from the center of the capillary after the fiber embedding. The deviation of the fiber from the capillary center can be used to predict the strain at the fiber’s real location.

### 3.4. Four-Point Bending Test Results of Al6082-T651

**Figure 15:**
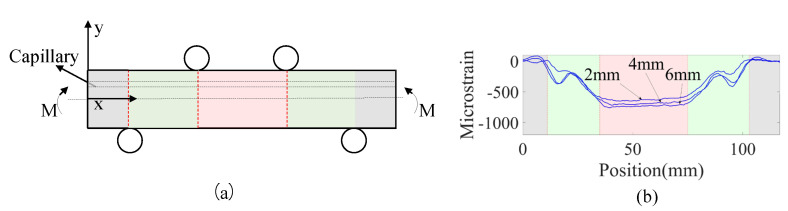
(**a**) The specimen under four-point bending fatigue test is divided into several sections, marked with different colors; (**b**) strain distribution measured by FOSs embedded within Specimens 01, 03, and 05 with capillaries of diameters 2 mm, 4 mm, and 6 mm, respectively, under the 8 KN loading under four-point bending static test.

Figure 15 presents the strain distribution measured by FOSs embedded within Specimens 01, 03, and 05 with capillaries of diameters 2 mm, 4 mm, and 6 mm, respectively, under the 8 KN loading under the four-point bending static test. It can be seen that the red region has a constant bending moment, which is of interest because the influence of the fiber locations and orientations can be highlighted, and the influence of the bending moment does not need to be considered in this region. The strain peaks in the green region are induced by the concentrated load from the loading pins in the four-point bending test.

## 4. Discussion

### 4.1. Comparison of the Analytically Predicted Strain, Numerically Predicted Strain and Measured Strain

**Figure 16:**
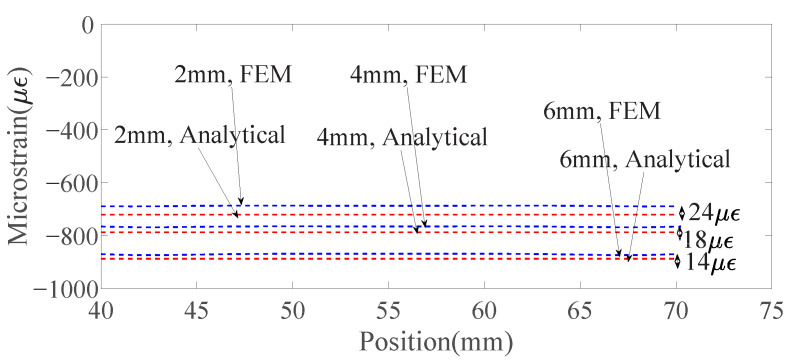
Comparison of the analytically predicted and numerically predicted strain distribution along the capillary center in the region with a constant bending moment for specimens under the loading of 8 KN.

**Figure 17:**
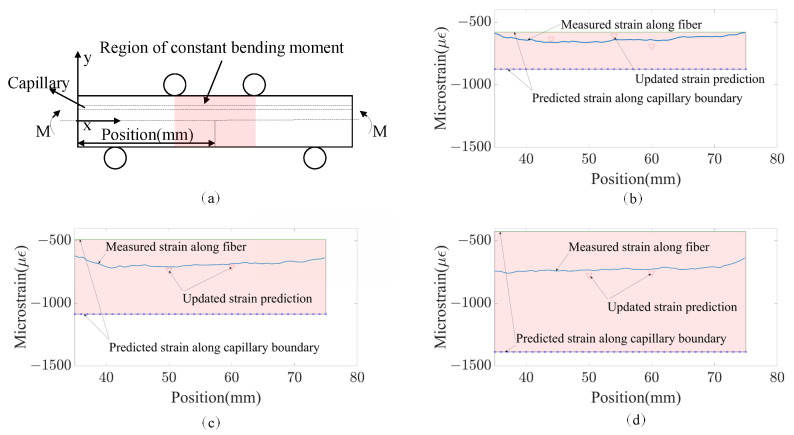
(**a**) Part of the capillary where bending moment is constant and is marked in red; comparison of strain measurement along with fiber embedded in the region marked red, and analytical strain predictions at the real fiber location when capillary diameters of Al6082 specimens are (**b**) 2 mm, (**c**) 4 mm and (**d**) 6 mm.

Figure 16 shows the comparison of the analytically predicted and numerically predicted strain distribution along the capillary center in the region with a constant bending moment for specimens under the loading of 8 KN. Figure 17 compares the strain measurement along with fiber embedded in the region marked with red, and analytical strain predictions at the real fiber location when capillary diameters of the Al6082 specimens are 2 mm, 4 mm, and 6 mm. It can be seen that the updated strain prediction with the real location of the fiber is very close to the measured strain. The difference between them is within the range of the measurement noise. In the analytical model, the capillary with the embedded fiber is directly modeled as an empty hole with zero stiffness, and the capillary in the numerical model is modeled as a capillary with adhesive resins. Due to the stiffness of the adhesive resins being much smaller than the substrate alloy and close to 0, the two models are therefore very similar in terms of modeling the capillary. In addition to that, the three basic assumptions of the Euler–Bernoulli beam theory are met. All of that can explain the small differences between the results from the analytical model and the numerical model.

Comparison of the analytical, numerical, and test results shows that violation of the analytical model assumption does not have an influence on the prediction accuracy of the analytical model. Since it is much easier to plot the axial strain along the fiber length when the fiber is wavy with the analytical model compared with the numerical model, the analytical model is necessary for investigating the influence of fiber orientations on the measured strain distributions. In addition, the results of the numerical model are also used in establishing the analytical model as shown in the subsequent section.

### 4.2. Influence of Fiber Position and Orientation Variations on Strain Measure Accuracy

**Figure 18:**
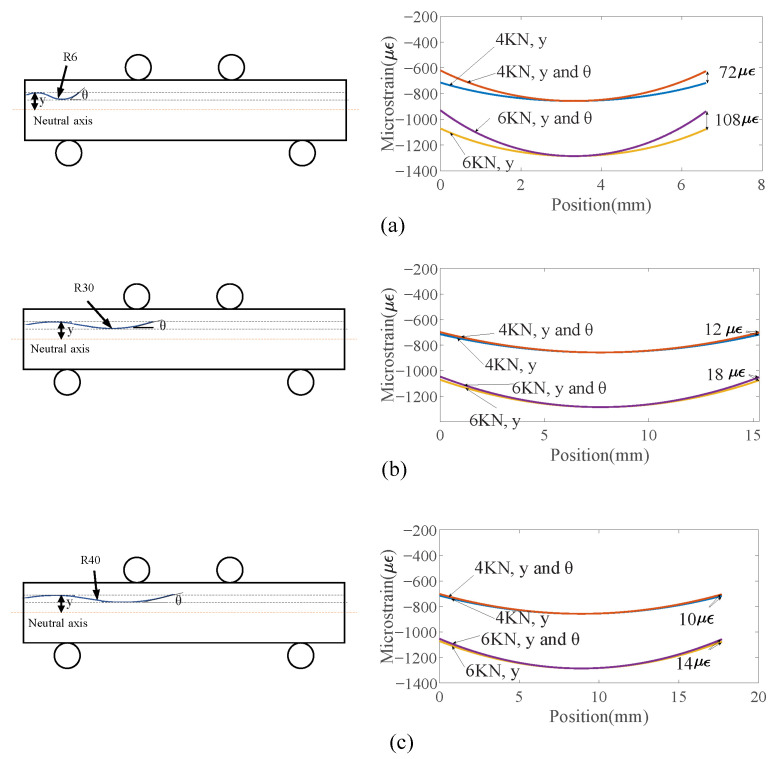
Comparison of analytically predicted strain along curved fiber when only fiber orientation θ and fiber position relative to neutral axis *y* are considered and θ alone are considered for curved fibers with a curvature radius of (**a**) 6 mm, (**b**) 30 mm, and (**c**) 40 mm, respectively, as shown on the left.

When an optical fiber is embedded within a specimen using capillaries, fiber positions with respect to the specimen centroid and fiber orientations may vary along the fiber. It is interesting to know whether both factors influence strain measurement, and how much their influences are. Analytical strain distribution along the fiber can be predicted using Equation (9). The right side of the equation indicates the influence of the fiber orientation on the strain measurement. When an optical fiber is embedded in a curved fiber with different arc-like shapes, the strain distributions, as a function of the fiber positions alone and as a function of both the fiber positions and orientations, are compared in Figure 18. When plotting the figure, the shear stress $\tau_{yx}$ in Equation (9) is from the results of the numerical model. It can be seen that when the fiber is curved to its limit of a 6 mm radius, the fiber orientations have an impact on the strain distribution. The maximum difference of the strain distribution when considering the fiber position relative neutral axis *y* and fiber orientation θ due to fiber bending is 90 με under 4 KN and 140 με under 8 KN. That is much greater than the measurement noise of the embedded FOSs. The measurement noise is determined by analyzing the measured strain distribution when there is not any loading on the specimen, and it is found that the measurement noise is between 0 and 20 με. Even though the differences of 90 με under 4 KN and 140 με under 8 KN are larger than the measurement noise, it is very rare to achieve this kind of waviness when embedding a fiber. As the fiber becomes less wavy, the difference decreases significantly. For example, when the fiber is in the arc-like shape with 30 mm, the maximum difference drops to 17 με, which is very close to the maximum measurement noise. Therefore, when embedding a fiber, in general, the fiber position relative to the neutral axis *y* has a much larger influence than the fiber orientation. The fiber orientation’s influence is not considered when using the measured strain for crack detection.

### 4.3. Relative Measurement Error Associated with Capillary

**Figure 19:**
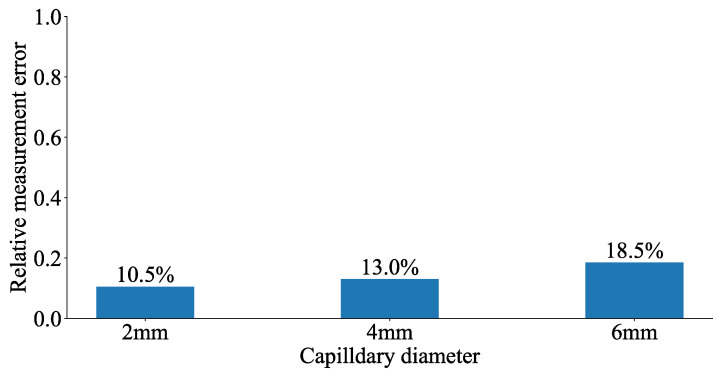
Measurement error of fibers embedded in capillaries of different diameters.

When assuming that the nominal strain distribution is the strain along the capillary center, variations of the fiber positions within the capillary lead to deviations of the measured strain from the nominal strain. The deviation can be quantified by relative measurement errors (RME), which can be defined by the following equation:

(13) $$RME=\frac{\left|\epsilon_{upper}-\epsilon_{lower}\right|}{2\epsilon_{center}},$$

where the $\epsilon_{upper}$, $\epsilon_{lower}$, and $\epsilon_{center}$ represent the strain value along the capillary upper boundary, lower boundary, and center. The strain measurements of a straight fiber deviate most from the nominal strain when the fibers are at the capillary boundaries, and the corresponding RMEs are the highest. Therefore, the capillary diameter has an influence on the relative measurement error of the embedded fiber. Figure 19 represents the RME for specimen capillaries, calculated with the analytically predicted strain, and it can be seen that as the capillary diameter increases, the fiber measurement error increases. That indicates that the fiber measurement accuracy decreases as the capillary diameter grows. Theoretically, the capillary diameter should be as small as possible in order to reduce the relative measurement error. However, reducing the capillary diameter also increases the difficulty of embedding fibers into the capillary because the adhesive resin should be injected into the capillary. Therefore, when the capillary is straight, the capillary diameter should be as small as possible to reduce the measurement error, and the ease of bonding is also an important factor to consider. In the experiment, the fiber can be easily embedded inside the capillary with a 2 mm diameter. However, when the capillary is wavy, it can be expected that a larger capillary diameter may be required to easily insert the fiber. That is because the brittle fiber can more easily break when inserted inside the wavy capillary.

## 5. Conclusions and Future Work

When an optical fiber is embedded within a specimen using capillaries, both the fiber positions and orientations may vary along the fiber. An analytical model was established to investigate the influence of fiber positions and variation change on strain distribution. The model was subsequently verified by the four-point bending static test. Based on the results of the analytical model and static four-point bending test, the following conclusions can be made.

1. When an optical fiber is embedded within a specimen using capillaries, both the fiber positions and orientations may vary along the fiber. Variations of the fiber orientations have a negligible influence on the strain measurement compared with fiber position variations. Therefore, fiber position uncertainty is the main factor influencing strain measurement accuracy.
2. Even the positional uncertainties of the embedded fiber within capillaries can cause strain measurement uncertainties; the measured strain distributions lie within the strain windows defined by the strain distribution along the capillary boundary when there are no cracks in the specimen. It is possible to perform crack detection using the strain window.

Because the measured strain distribution always lies within the strain window when there are no cracks in the specimen, it is possible to compare the measured strain distribution against the strain window. Any exceedance of the measured strain beyond the strain window indicates the existence of an anomaly, such as a crack. It is possible to perform crack detection based on this principle, which will be studied in a follow-up study.

## 6. Source Code

The code for building the analytical model can be seen in the GitHub repository [https://github.com/Yuzhe17/Measurement-accuracy-of-embedded-fiber-optic-sensors.git, (accessed on 17 January 2023)]. 

## Figures and Tables

**Table 1:** Mechanical properties of the specimen and adhesive resin.

Material	Elastic Modulus (GPa)	Yield Strength (MPa)	Poisson’s Ratio
Aluminum 6082-T651	70	270	0.33
AW106/HV953U epoxy adhesives	1.9 [17]	-	-

**Table 2:** Number and dimensions of all the specimens.

Specimen ID	Specimen Material	Capillary Diameter d [mm]	Specimen Length L [mm]	Specimen Width b [mm]	Specimen Height h [mm]
01	Aluminum 6082-T651	2	110	12	20
02	Aluminum 6082-T651	2	110	12	20
03	Aluminum 6082-T651	2	110	12	20
04	Aluminum 6082-T651	4	110	12	20
05	Aluminum 6082-T651	4	110	12	20
06	Aluminum 6082-T651	4	110	12	20
07	Aluminum 6082-T651	6	110	12	20
08	Aluminum 6082-T651	6	110	12	20
09	Aluminum 6082-T651	6	110	12	20

**Table 3:** Different test cases for the static four-point bending test.

Test Case No.	Specimen ID	Specimen Capillary Diameter (mm)
1	01	2
2	02	2
3	03	4
4	04	4
5	05	6
6	06	6

## Data Availability

The data presented in this study are available on request from the corresponding author.
